# Comparison of Phytochemicals, Antioxidant, and In Vitro Anti-Alzheimer Properties of Twenty-Seven *Morus* spp. Cultivated in Thailand

**DOI:** 10.3390/molecules25112600

**Published:** 2020-06-03

**Authors:** Piya Temviriyanukul, Varittha Sritalahareuthai, Kriskamol Na Jom, Butsara Jongruaysup, Somying Tabtimsri, Kanchana Pruesapan, Sirinapa Thangsiri, Woorawee Inthachat, Dalad Siriwan, Somsri Charoenkiatkul, Uthaiwan Suttisansanee

**Affiliations:** 1 Institute of Nutrition, Mahidol University, Salaya, Phuttamonthon, Nakhon Pathom 73170, Thailand; piya.tem@mahidol.ac.th (P.T.); varittha.sri@hotmail.com (V.S.); poo.sweet@hotmail.com (S.T.); woorawee.int@mahidol.ac.th (W.I.); somsri.chr@mahidol.ac.th (S.C.); 2 Department of Food Science and Technology, Faculty of Agro-Industry, Kasetsart University, Chatuchak, Bangkok 10900, Thailand; kriskamol.n@ku.ac.th; 3 Office of Sericulture Conservation and Standard Conformity Assessment, The Queen Sirikit Department of Sericulture, Ministry of Agriculture and Cooperatives, Bangkok 10900, Thailand; butsara_2000@hotmail.com; 4 The Queen Sirikit Department of Sericulture Center (Kanchanaburi), Nong Ya, Mueang Kanchanaburi District, Kanchanaburi 71000, Thailand; yodyingtts@gmail.com; 5 Plant Varieties Protection Office, Department of Agriculture, Ministry of Agriculture and Cooperatives, Bangkok 10900, Thailand; kpruesapan@gmail.com; 6 Institute of Food Research and Product Development, Kasetsart University, Chatuchak, Bangkok 10900, Thailand; dalad.s@ku.th

**Keywords:** *Morus* species, anthocyanins, anthocyanidins, beta-secretase 1, antioxidant, anti-Alzheimer properties

## Abstract

Alzheimer’s disease (AD) is a progressive neurodegenerative disorder. To fight the disease, natural products, including mulberry, with antioxidant activities and inhibitory activities against key enzymes (acetylcholinesterase (AChE), butyrylcholinesterase (BChE), and beta-secretase 1 (BACE-1)) are of interest. However, even in the same cultivars, mulberry trees grown in different populated locations might possess disparate amounts of phytochemical profiles, leading to dissimilar health properties, which cause problems when comparing different cultivars of mulberry. Therefore, this study aimed to comparatively investigate the phytochemicals, antioxidant activities, and inhibitory activities against AChE, BChE, and BACE-1, of twenty-seven *Morus* spp. cultivated in the same planting area in Thailand. The results suggested that *Morus* fruit samples were rich in phenolics, especially cyanidin, kuromanin, and keracyanin. Besides, the aqueous *Morus* fruit extracts exhibited antioxidant activities, both in single electron transfer (SET) and hydrogen atom transfer (HAT) mechanisms, while strong inhibitory activities against AD key enzymes were observed. Interestingly, among the twenty-seven *Morus* spp., *Morus* sp. code SKSM 810191 with high phytochemicals, antioxidant activities and in vitro anti-AD properties is a promising cultivar for further developed as a potential mulberry resource with health benefits against AD.

## 1. Introduction

Aging is recognized as the irreversible and inevitable loss of physiological integrity, leading to aging-associated disorders, including cardiovascular disease, cancer, metabolic syndrome, and neurodegenerative diseases [1]. Further studies have also shown that aging increases the decline of nervous system functions. Alzheimer’s disease (AD), a devastating type of dementia, is associated with aging, as approximately 90% of AD cases are found in individuals older than 65 years. It has been suggested that there will be 131 million AD cases by 2050 [2]. However, there are only five drugs approved for AD treatment, including four cholinesterase inhibitors (tacrine, rivastigmine, galantamine, and donepezil) and one glutaminergic *N*-methyl-D-aspartate (NMDA) receptor antagonist (memantine) [3]. Cholinesterase inhibitors have been shown to inhibit the enzymatic functions of cholinesterases, including acetylcholinesterase (AChE) and butyrylcholinesterase (BChE), resulting in the accumulation of a neurotransmitter, acetylcholine, at the synaptic cleft. Meanwhile, memantine blocks the effects of glutamate, which is over-stimulated in AD patients [3]. In addition, an alternative AD treatment has also been intensively studied. Attention has shed light on the beta-secretase 1 (BACE-1) inhibitors because BACE-1 is an enzyme involved in the formation of amyloid or senile plaques, a hallmark of AD. Intriguingly, several reports have highlighted the effective BACE-1 inhibitory activities from plant extracts, such as ursolic acid and lupeol from *Leea indica* [4], polymethoxyflavones, 5,7-dimethoxyflavone (DMF), 5,7,4’-trimethoxyflavone (TMF), and 3,5,7,3’,4’-pentamethoxyflavone (PMF) from *Kaempferia parviflora* [5], and moracin derivatives from *Morus* species [6].

Mulberry fruits in *Morus* species, belonging to the Moraceae family, are widely distributed in tropical, sub-tropical, and sub-arctic regions, including Europe, Asia, America, and Africa, suggesting that *Morus* spp. is able to adapt to various varieties of climatic and soil conditions [7]. At present, this genus consisting of twenty-four species and one-hundred varieties is suspected to have different health benefits [7]. *Morus* spp. has long been cultivated and used for sericulture, food, and folk medicine. Mulberry fruits are low in calories with a sour taste (pH ≤ 3.5) and high in phytochemicals, predominantly anthocyanins [8]. Anthocyanins, a member of flavonoids, are responsible for red or purple pigments in vegetables and fruits. The natural-occurring anthocyanins in the plants are in the form of glycosides (binding to carbohydrate moieties), while those without the carbohydrate moieties are subsequently called anthocyanidins [9]. It has been reported that the distribution of anthocyanins in vegetables and fruits is cyanidin (50%), delphinidin (12%), pelargonidin (12%), peonidin (12%), malvidin (7%), and petunidin (7%), while the most common form of the glycoside derivative in nature is cyanidin 3-glucoside [10]. Anthocyanins exhibit health benefits against a range of ailments, including oxidation, cancer, anemia, obesity, diabetes, hypertension, and AD activities [8]. Given the good taste and health benefits of *Morus* spp., the cultivation and consumption of mulberry fruits have been swiftly developed around the word, including Thailand.

There are more than thirty mulberry varieties registered by the Queen Sirikit Department of Sericulture, Ministry of Agriculture and Cooperatives, Thailand. However, only a few cultivars have been investigated for their phytochemical profiles and health benefits. Furthermore, regarding the high adaptation of *Morus* spp. that can be cultivated in several regions of the world, as mentioned above, the *Morus* spp. cultivated in different areas, even from the same cultivars, might possess different amounts of phytochemical profiles and health properties, leading to difficulty in comparing these parameters. Therefore, this study was undertaken to evaluate the phytochemical profiles (total phenolic, anthocyanin, and anthocyanidin contents), and health properties (antioxidant and anti-Alzheimer properties) of twenty-seven *Morus* spp. cultivated in the same planting area in Kanchanaburi province, Thailand. This area belongs to the *Morus* spp. genetic bank project of the Ministry of Agriculture and Cooperatives, Thailand. This is the first study that has provided comparative and comprehensive data of twenty-seven *Morus* spp., which could indorse further development of the phytochemical compound rich mulberry resources with potential health benefits against the occurrence of AD.

## 2. Results

### 2.1. Total Phenolic Contents (TPCs), Anthocyanins and Anthocyanidins

**Figure 1:**
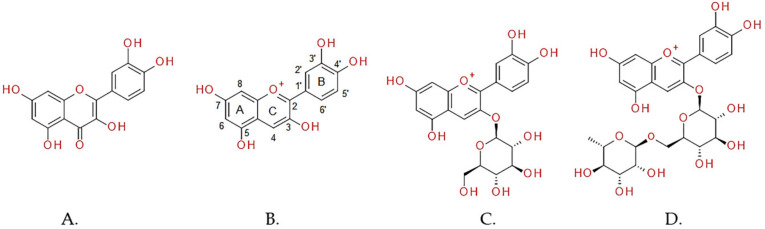
Chemical structures of (**A**) quercetin, (**B**) cyanidin, (**C**) kuromanin (cyanidin 3-glucoside), and (**D**) keracyanin (cyanidin 3-rutinoside).

The total phenolic contents (TPCs) of *Morus* fruit extracts were within the range of 0.37–11.86 mg GAE/g dry weight (DW), with *Morus* sp. code SKSM 810391 extract exhibiting the highest TPCs, and *Morus* sp. code SKSM 810391 extract providing the lowest (Table 1). The only anthocyanidin in *Morus* fruit extracts, as determined by HPLC analysis, was detected as cyanidin (Figure 1) ranging between 41.25 and 2879 µg/g DW (Table 1 and Supplementary Figure S1). *Morus* sp. code SKSM 810191 extract possessed the highest content of cyanidin, while *Morus* sp. code SRCM 9809-34 extract exhibited the lowest. However, none was detected in *Morus* sp. code SRCM 9124-12 extract. Two anthocyanins, including keracyanin (cyanidin 3-glucoside) and kuromain (cyanidin 3-rutinoside) (Figure 1), were detected in the range of 121.3–7588 and 88.50–13566 µg/g DW, respectively (Table 1 and Supplementary Figure S2). The highest keracyanin and kuromanin contents were detected in *Morus* sp. code SKSM 810191 extract, while the lowest amount was found in *Morus* sp. code SRCM 9801-535 extract for keracyanin and *Morus* ‘Nakhon Ratchasima 60’ extract for kuromanin. However, neither keracyanin nor kuromanin was detected in *Morus* sp. code SKSM 040691 extract.

### 2.2. Antioxidant Activities

Antioxidant activities of *Morus* fruit extracts were determined using 2,2-diphenyl-1-picrylhydrazyl (DPPH) radical scavenging activity, ferric ion reducing antioxidant power (FRAP), and oxygen radical absorbance capacity (ORAC) assays. The difference among these methods involved the reaction mechanisms, in which DPPH radical scavenging and FRAP assays presented the mechanism of single electron transfer (SET), while ORAC assay underwent the hydrogen atom transfer (HAT) mechanism [11]. Since various species of oxidants (i.e., reactive oxygen species and reactive nitrogen species) were generated, more than one assay for detecting antioxidant activities was suggested to appropriately interpret the antioxidant results.

The results (Table 2) suggested that all *Morus* fruit extracts exhibited scavenging activities of 0.28–1.25 µmol TE/100 g DW with *Morus* ‘Kun Pai’ extract exhibiting the highest activity and *Morus* ‘Nakhon Ratchasima 60′ extract the lowest. The chelating abilities of ferrous ion ranged between 2.30 and 117.8 µmol TE/g DW, as investigated by the FRAP assay. The highest chelating ability was found in *Morus* sp. code SKSM 810391 extract, while the lowest was in *Morus* ‘Pai’ extract. Antioxidant capacity measured by the ORAC assay ranged between 64.03 and 283.2 µmol TE/g DW. *Morus* sp. code SKSM 810191 extract possessed the highest ORAC activity, while *Morus* sp. code SRCM 9124-12 extract exhibited the lowest.

### 2.3. In Vitro Cholinesterase and BACE-1 Inhibitory Activities

*Morus* fruit extracts were able to inhibit the key enzymes involved in AD, including AChE, BChE, and BACE-1, with different degrees of inhibition (Table 3). The AChE inhibitory activities of all *Morus* fruit extracts were in the range of 21.87–60.09% inhibition at the extract concentration of 5 mg/mL. *Morus* ‘Nakhon Ratchasima 60′ extract exhibited the highest AChE inhibitory activity, while *Morus* sp. code SKSM 810391 extract exhibited the lowest. Under the same extract concentration, BChE inhibitory activities ranged between 21.27% and 77.02% inhibition, with *Morus* ‘Som’ extract exhibiting the highest inhibition and *Morus* sp. code SRCM 9801-465 extract the lowest. Likewise, *Morus* fruit extracts exhibited BACE-1 inhibitory activity in the range of 31.28–78.67% inhibition at the extract concentration of 5 mg/mL. *Morus* sp. code SRCM 9806-287 extract exhibited the highest BACE-1 inhibition, while the lowest inhibition was detected in *Morus* sp. code SRCM 9124-12 extract.

### 2.4. Correlation Analysis of Bioactive Compounds, Antioxidant Activities and AD Key Enzyme Inhibitory Activities

The relationship among bioactive compounds, antioxidant activities and enzyme inhibitory activities was reported as correlation coefficients (*r*) between values (Table 4). The *r* ranges were divided into three groups; weak correlation (*r* = −/+0.10 to −/+0.29), moderate correlation (*r* = −/+0.30 to −/+0.49) and strong correlation (*r* = −/+0.50 to −/+1.0). The results among phytochemical compounds suggested a strong positive correlation between TPCs and the contents of keracyanin (*r* = 0.540) and kuromanin (*r* = 0.505), while a moderate positive correlation between TPCs and cyanidin contents was detected (*r* = 0.476). Moreover, strong positive correlations among cyanidin, keracyanin and kuromanin were observed (*r* = 0.943–0.977). The relationship between phytochemical compounds and antioxidant activities suggested a strong positive correlation between TPCs and DPPH radical scavenging activities (*r* = 0.502), while forming a moderate positive correlation with ORAC activities (*r* = 0.421) and a weak positive correlation with FRAP activities (*r* = 0.242). On the other hand, keracyanin formed strong positive correlations with antioxidant activities determined by FRAP (*r* = 0.678) and ORAC assays (*r* = 0.626). Similar results were observed with strong positive correlations between cyanidin and kuromanin with all three methods of antioxidant measurement (*r* = 0.527–0.772 for cyanidin and 0.498–0.725 for kuromanin). Nevertheless, weak to moderate correlations between the amounts of phytochemicals compounds and inhibitory activities of AD key enzymes were observed with *r* ranging from −0.305 to 0.416. Likewise, weak to moderate correlations between antioxidant activities and inhibitory activities of AD key enzymes were reported with *r* ranging from −0.480 to 0.300. Interestingly, AChE inhibitory activities formed a strong correlation (*r* = 0.860) with BChE inhibitory activities, while no correlations were observed between inhibitory activities of cholinesterase and BACE-1 (*r* ranged from −0.037 to −0.020).

**Figure 2:**
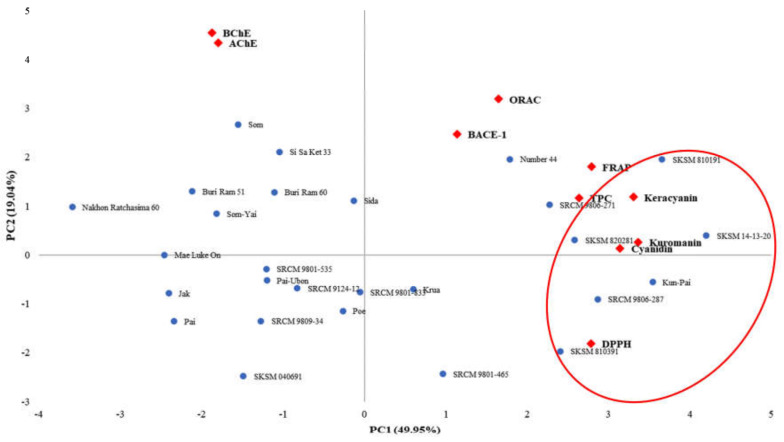
Biplot of principal component analysis from mean value of all variables (●) investigated in twenty-seven *Morus* cultivars (♦).

**Figure 3:**
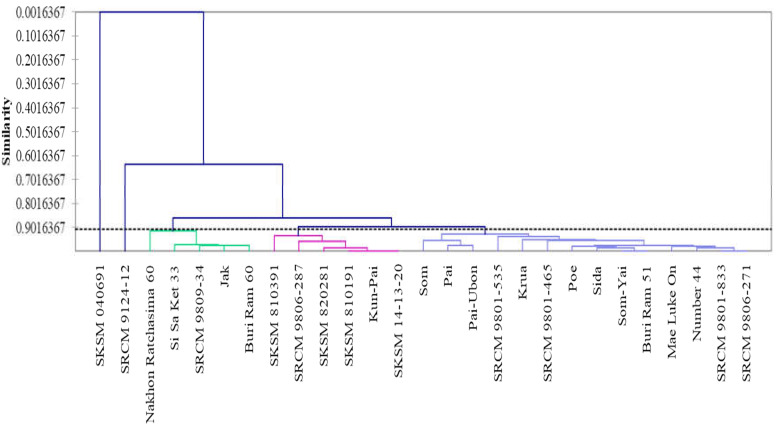
Dendrogram (similarity mode of agglomerative hierarchical clustering analysis) of twenty-seven *Morus* cultivars by mean value of all variables.

To independently analyze the correlation among bioactive compounds, antioxidant activities and enzyme inhibitory activities, mean values of all variables, including phenolic contents (TPCs, cyanidin, keracyanin, and kuromanin), antioxidant activities (DPPH radical scavenging, FRAP, and ORAC values), and inhibitory activities of AD key enzymes (AChE, BChE, and BACE-1), obtained for twenty-seven *Morus* cultivars were subjected to statistical analysis via principal component analysis (PCA) to verify if the mulberry cultivars could be differentiated according to the mentioned variables. A PCA biplot (Figure 2) showed that differentiation among twenty-seven *Morus* cultivars shifted along the PC1 and PC2 axes representing ~65% of total variables. TPCs, anthocyanins, anthocyanidin, antioxidant activities and BACE-1 inhibitory activities were clustering together. Furthermore, agglomerative hierarchical clustering analysis (AHC) in similarity mode was performed as shown in Figure 3 and resulted in twenty-seven *Morus* cultivars being divided into four groups. Interestingly, both PCA biplot and the dendrogram showed that a group of six cultivars, including *Morus* sp. code SKSM 810191, SKSM 810391, SKSM 820281, SKSM 14-13-20, SRCM 9806-287 and *Morus* ‘Kun Pai’, was gathered together based on their high phytochemicals, antioxidant activities and BACE-1 inhibitory activities.

## 3. Discussion

There is evidence demonstrating that plant extracts possess potential health benefits against AD through several mechanisms underlying AD pathogenesis, particularly by the reduction of oxidative stress, cholinesterases, and BACE-1 activities. In addition to their efficacy, plant extracts also seem to be safer than synthetic drugs. *Morus* spp. are of great interest due to their high phytochemicals, especially anthocyanins and anthocyanidins, which have been proved to exhibit anti-AD functions in vitro and in vivo [12]. Besides anthocyanins and anthocyanidins, moracin derivatives from *Morus radix* could function as dual BACE1 and cholinesterase inhibitors with antioxidant and anti-glycation capacities [6]. In Thailand, more than thirty mulberry varieties are planted under the *Morus* spp. genetic bank project of the Ministry of Agriculture and Cooperatives. However, the phytochemical profiles and health properties of these mulberry fruits are still missing. Therefore, this is the first comparative and comprehensive study of twenty-seven *Morus* spp. regarding their phytochemical profiles (total phenolic contents, as well as anthocyanins and anthocyanidins contents), and health properties (anti-oxidant and anti-Alzheimer properties). We have found that (i) *Morus* fruit samples were rich in phenolics, anthocyanidin (cyanidin) and anthocyanins (kuromanin and keracyanin), (ii) aqueous *Morus* fruit extracts are involved in anti-oxidative stress, both in single electron transfer (SET) and hydrogen atom transfer (HAT) mechanisms, (iii) aqueous *Morus* fruit extracts exhibited strong inhibitory activities against AD key enzymes (AChE, BChE and BACE-1), and (vi) *Morus* sp. code SKSM 810191 provided high TPCs, anthocyanins and anthocyanidin contents, antioxidant activities and in vitro anti-AD properties, which can be further developed as a potential mulberry resource with health benefits against AD.

It was previously suggested that colors (cultivars) of mulberry yielded a great impact on their bioactive compounds [7,13,14,15]. Juice of white (*M. alba* L.), red (*M. rubra* L.), and black (*M. nigra* L.) mulberry fruits from Turkey suggested that black mulberry exhibited the highest TPCs (1422 mg GAE/100 g fresh weight (FW)), followed by red (1035 mg GAE/100 g FW) and white mulberries (181 mg GAE/100 g FW), respectively [7]. These data corresponded to our study, in which the TPCs of the aqueous *Morus* fruit extracts (purple-red color) ranged between 71 and 2270 mg GAE/100 FW (or 0.37–11.86 mg GAE/g DW). Besides, the TPCs of the aqueous ethanolic extracted *M. alba* from Korea (the TPCs of 0.96–2.57 mg GAE/g DW) [16] and the methanolic extracted *M. alba* from North Serbia (the TPCs of 1.05–2.16 mg GAE/g DW) [17] were in the same range as the TPCs of our aqueous *Morus* fruit extracts (0.37–11.86 mg GAE/g DW). As for anthocyanidins, cyanidin is the most abundant anthocyanidin (50%) detected in fruits and vegetables, followed by delphinidin (12%), pelargonidin (12%), peonidin (12%), malvidin (7%), and petunidin (7%) [18]. Cyanidin gives a magenta color; thus, it is mostly found in reddish-purple berries or vegetables [19]. In our experiment, cyanidin (41.25–2879.30 µg/g DW) was the only anthocyanidin detected in all *Morus* fruit extracts, while delphinidin, pelargonidin, peonidin and petunidin were undetected. Besides, two glycosides of cyanidin, keracyanin (cyanidin 3-rutinoside, 121.3–7,588 µg/g DW,) and kuromanin (cyanidin 3-glucoside, 88.50–13,566 µg/g DW), were found in our *Morus* fruit extracts. These results corresponded to the previous studies, which suggested that the predominant anthocyanins found in mulberry (*M. alba* L.) extracted with acidic ethanol were keracyanin (60%) and kuromain (38%), while traces of pelargonidin 3-*O*-glucoside and pelargonidin 3-*O*-rutinoside were also detected at 2% in total [20]. Previous studies also suggested that aqueous ethanolic extracts of five cultivars of Korean mulberry (*M. alba* L.) exhibited keracyanin, ranging between 30.6 and 486.7 µg/g DW, and kuromanin ranging between 93.2 and 1364.9 µg/g DW [16], which were in the ranges of our anthocyanin contents. Other than these major anthocyanins, Chinese mulberry (*M. alba* L.) extracted with acidic methanol and defatted with ethyl acetate was found to exhibit five anthocyanins, including cyanidin 3-*O*-(6″-*O*-α-rhamnopyranosyl-β-d-glucopyranoside) (C3RG), cyanidin 3-*O*-(6′′-*O*-arhamnopyranosyl-β-d-galactopyranoside) (C3RGa), cyanidin 3-*O*-β-d-glucopyranoside (C3G), cyanidin 3-*O*-β-d-galactopyranoside (C3Ga) and cyanidin 7-*O*-β-d-glucopyranoside (C7G) [21]. Brazil wild mulberry (*M. nigra* L.) extracted under saponification and acid hydrolysis consisted of kuromanin (71%) and cyanidin 3-glucosylrhamnoside (19%) [22]. Therefore, types and quantities of detected anthocyanins/anthocyanidins depended on both internal factors (i.e., cultivars, fruit color, and stages of maturity) and external factors (i.e., climate, growing location, and extraction methods).

One of the biological functions of phenolics is that of anti-oxidative agents. The strong positive correlation between TPCs and DPPH radical scavenging activities, a moderate positive correlation with ORAC activities, and a weak positive correlation with FRAP activities suggested that most phenolics detected in *Morus* fruit samples are able to scavenge free radicals in the SET mechanism. On the other hand, keracyanin with strong positive correlations to antioxidant activities determined by FRAP and ORAC assays indicated that keracyanin could function as an antioxidant in both SET and HAT mechanisms. Likewise, the strong positive correlations between cyanidin and kuromanin with all three methods of antioxidant measurement suggested that these phytochemical compounds could behave as antioxidants in both SET and HAT mechanisms. The SET mechanism related to antioxidants that are able to transfer electron (electron donor) to any electron acceptors such as metals, carbonyls, and radicals. The examples of antioxidant capacity measurements under this mechanism are DPPH radical scavenging and FRAP assays [23]. In DPPH radical scavenging assay, the deep blue DPPH^•^ radical reacts with an antioxidant to produce a yellow DPPH–H product. The FRAP assay, however, involves the ability to reduce brown ferric (Fe^3+^) to indigo ferrous (Fe^2+^) ions in the presence of Fe^2+^–stabilizing ligand such as 2,4,6–tripyridyl–*s*–triazine (TPTZ). The HAT mechanism is based on the ability of antioxidants to quench free radicals by hydrogen atom donation. The example of this mechanism is ORAC assay, in which antioxidant capacity is demonstrated from the kinetic curves based on competitive inhibition of chemical kinetics [23]. The peroxyl radical generated from thermogenesis of AAPH (oxidizing agents) can react with a fluorescein probe to produce non-fluorescent fluoresceinyl radicals, while antioxidants of interest are acting as competitive inhibitors, and antioxidant activity can be measured. To appropriately interpret the antioxidant actions, more than one assay is usually performed to investigate the antioxidant capacities. It was previously suggested that the antioxidant activities of anthocyanins/anthocyanidins are diverse, according to types of reactive species, environments (i.e., pH, heat, and light exposure), and anthocyanins/anthocyanidins structures. For types of reactive species, it was found that pelargonidin is the strongest hydroxyl radical scavenger, followed by cyanidin and delphinidin, respectively [24]. However, opposite results were observed with superoxide anion scavenging capacity, in which delphinidin is the strongest scavenger, followed by cyanidin and pelargonidin, respectively [24]. The effect of the environment is related to sensitivity of detected anthocyanins/anthocyanidins, which also depend on extraction methods, including pH, temperature, and light exposure [20]. Interestingly, the degree and position of hydroxyl, methoxyl, and sugar moieties of anthocyanins/anthocyanidins play a significant role in their antioxidant capacities. It was previously suggested that the increased number of free hydroxyl moieties around a pyrone ring on anthocyanin/anthocyanidin structures can elevate antioxidant capacity [25]. Besides, the presence of 3’,4’-dihydroxyl groups on the B ring (Figure 1) promotes the metal ions chelating reaction [26], while the presence of dihydroxyl moieties on the ortho-positions around the C4’ position on the B ring positively affect the scavenging activity of hydroxyl radicals through iron chelation [27]. The presence of methoxyl moieties, however, reduces antioxidant capacity [25,28]. The addition of methoxyl moiety at the 5’ position on the B ring (petunidin) can decrease radical scavenging activity 3-fold, compared to the one without (cyanidin) [25]. Additionally, glycosylation processes, including a number of sugar residues, types and positions of sugar, and types of glycosidic bond on anthocyanidins, vary the stability of anthocyanins, leading to different antioxidant capacities [25,29]. Interestingly, the increased number of sugar moieties at the C3 position on the C ring of anthocyanins (Figure 1) can decrease antioxidant activity [30]. It was previously reported that the radical scavenging activities of cyanidin were higher than kuromanin and keracyanin, respectively [25]. These results corresponded to our findings, in which cyanidin and kuromanin contents were strongly correlated to DPPH radical scavenging values, while the correlation between keracyanin and DPPH radical scavenging activity was moderated.

Not only were aqueous *Morus* fruit extracts involved in anti-oxidative stress, but they also exhibited strong inhibitory activities against AD key enzymes (AChE, BChE and BACE-1). The AChE inhibition ranging between 21.87% and 60.09% and the BChE inhibition between 21.27% and 77.02% of our aqueous *Morus* fruit extracts (5 mg/mL) are probably the results of the biological function of anthocyanins/anthocyanidins. Cyanidin was previously found to exhibit the IC_50_ of 14.43 µM against AChE and a slightly higher IC_50_ value for BChE inhibition [31]. However, its glycosylated forms, keracyanin and kuromanin, exhibited lower AChE and BChE inhibition [31]. To understand the molecular mechanism of how anthocyanins/anthocyanidins inhibit cholinesterase, previous studies performed molecular docking to investigate the interactions between the enzymes and the inhibitors. Even though the molecular mechanism on AChE inhibitory interactions between the enzyme and anthocyanins/anthocyanidins is unavailable, the study on quercetin with a similar chemical structure to cyanidin (Figure 1) suggests that the inhibition occurs through blockage of the active site entrance [32]. The number of hydroxyl moieties on flavonoids seems to increase the degree of inhibition [32]. In parallel to the AChE–quercetin interactions, it is highly possible that the hydroxyl moieties at the C3 position in the C ring and at the C5 position in the A ring of cyanidin can interact with the enzyme residues in the catalytic pocket of AChE. Besides, the hydroxyl moieties at the C3’ and C4’ positions in the B ring interact with the enzyme residues in the cavity entrance of AChE. Interestingly, the glycosylation on the C3 hydroxyl moiety in the C ring decreases inhibitory activity [32]. In addition, although it is unclear whether anthocyanins/anthocyanidins exhibit BACE-1 inhibitory activity, the molecular docking study showed that cyanidin could bind to the active site of BACE-1 better than that of a well-known BACE-1 inhibitor (BACE-1 inhibitor-IV) [33]. Together, the molecular docking analysis implies that anthocyanins/anthocyanidins in aqueous *Morus* fruit extracts may be responsible for cholinesterase and BACE-1 inhibition.

In conclusion, among the twenty-seven mulberries used in the study, *Morus* sp. code SKSM 810191 exhibited high TPCs, anthocyanins and anthocyanidin contents, antioxidant activities, and AD key enzyme inhibitions, highlighting its potential for phytochemical compounds of a rich mulberry resource with health benefits against AD occurrence.

## 4. Materials and Methods

### 4.1. Mulberry Collection, Preparation, and Extraction

The fruits of uniform color and ripening stage of twenty-seven *Morus* spp. were collected from the Queen Sirikit Department of Sericulture Center (Kanchanaburi), Thailand. The cultivars, sample code, and collector are listed in Supplementary Table S1. Mulberry fruits were cleaned once with tap water and twice with deionized water (DI). After that, the samples were freeze-dried (Heto PowerDry PL9000, Heto Lab Equipment, Allerød, Denmark) for 3 days. Dry samples were ground into fine powder using a grinder (Philips 600W series, Philips Electronics Co., Ltd., Jakarta, Indonesia), and packed in vacuum aluminum foil bags before extraction. The aqueous extract was then prepared, as previously described [12].

The colors of both fresh and dry samples were measured using a spectrophotometer (ColorFlex EZ, Hunter Associates Laboratory, Virginia, USA) and expressed as Hunter-Lab units, including L representing dark (0) to white (100) colors, a representing green (−) to red (+) colors and b representing blue (−) to yellow (+) colors. The moisture contents of powdery samples were analyzed using a Halogen moisture analyzer (HE53 series, Mettler-Toledo AG, Greifensee, Switzerland). The data of colors and moisture contents are showed in Supplementary Table S2.

### 4.2. Evaluation of Antioxidant Activity

The antioxidant activities including 2,2-diphenyl-1-picrylhydrazyl (DPPH) radical scavenging activity, oxygen radical absorbance capacity (ORAC), and ferric ion reducing antioxidant power (FRAP) assays of *Morus* fruit extracts, were performed using the well-established protocols indicated in Thuphairo et al. 2019 [34,35,36,37].

### 4.3. Determination of Total Phenolic Contents, Anthocyanin and Anthocyanidin

Total phenolic contents (TPCs) of *Morus* fruit extracts were determined using Folin–Ciocalteu reagent, as described by Thuphairo et al., 2019 [38]. Gallic acid (10–200 µg/mL) was used as a standard, and the TPCs were reported as mg gallic acid equivalent (GAE)/g dried matter (DW) [38].

To determine anthocyanidins, *Morus* fruit powder was extracted using acid hydrolysis by dispersion of the powdered sample (500 mg) in 50% (*v*/*v*) aqueous methanol containing 2 N HCl (5 mL). The extract was incubated in a 100 ± 2 °C water bath (TW20 series from Julabo GmbH, Seelbach, Germany) for 1 h and filtered through a 0.22 µM PTFE membrane syringe filter into a 2 mL HPLC vial. The identification of anthocyanidins of *Morus* fruit extracts (20 µL) was achieved by an UtiMate 3000 HPLC system with diode array and multiple-wavelength detectors from Thermo Fisher Scientific (Dreieich, Germany) utilizing a 5 µm ReproSil-Pur^®^ ODS-3 column (250 × 4.6 mm) from Dr. Maisch GmbH (Ammerbuch, Germany). Milli-Q water (18.2 MΩ.cm conductivity) containing 0.4% (*v*/*v*) TFA (solvent A) and acetonitrile containing 0.4% *v*/*v* TFA (solvent B) were used as isocratic mobile phase at 82% solvent A and 18% solvent B with a constant flow rate of 1.0 mL/min over 60 min. The anthocyanidins was visualized at 530 nm using a Chromeleon^TM^ Chromatography Data System (CDS) software (Thermo Fisher Scientific, Dreieich, Germany). The retention times (*R_t_*) and UV-Vis spectral fingerprints of standards including cyanidin (≥96.0% HPLC), delphinidin (≥97.0% HPLC), pelargonidin (≥97.0% HPLC), peonidin (≥97.0% HPLC), and petunidin (≥95.0% HPLC) from Extrasynthese (Genay, France) were used to confirm the existence of the anthocyanidins in *Morus* fruit extracts.

For anthocyanin analysis, *Morus* fruit extracts were prepared similarly to those for anthocynidins. However, low concentration of acid (2% (*v*/*v*) HCl in 5 mL of 50% (*v*/*v*) aqueous methanol) was applied to the extraction to stabilize anthocyanins. The HPLC analysis was performed utilizing a constant flow rate of 1 mL/min at ambient temperature. The solvent system was shown in Table 5 as previously described [12].

The existence of the anthocyanins was visualized at 525 nm and compared *R_t_* and UV-Vis spectral fingerprints with standards including callistephin (pelargonidin 3-*O*-glucoside) (≥95.0% HPLC), cyanidin 3-*O*-sophoroside (≥95.0% HPLC), cyanin (cyanidin 3,5-di-*O*-glucoside) (≥97.0% HPLC), delphin (delphinidin 3,5-di-*O*-glucoside) (≥97.0% HPLC), ideain (cyanidin 3-*O*-galactoside) (≥97.0% HPLC), keracyanin (cyanidin 3-*O*-rutinoside) (≥96.0% HPLC), kuromanin (cyanidin 3-*O*-glucoside) (≥96.0% HPLC), malvidin (*malvidin* 3-*O*-beta-d-glucoside) (≥97.0% HPLC), and pelargonidin 3-*O*-rutinoside (≥90.0% HPLC) from Extrasynthese (Genay, France).

### 4.4. Determination of Cholinesterases and Beta-secretase 1 (BACE-1) Inhibitory Activities

Cholinesterases (AChE and BChE) inhibitory activities of *Morus* fruit extracts were performed as previously reported [38,39,40]. Briefly, the enzyme assay consisting of 20 ng of *Electrophorus electricus* AChE (1000 units/mg, 100 μL) in 50 mM KPB (pH 7.0), 16 mM 5,5-dithio-bis-(2-nitrobenzoic acid) (DTNB, 10 μL), 0.8 mM acetylthiocholine (40 μL) in 50 mM KPB (pH 7.0), and the extract (50 μL) was detected at 412 nm using a microplate reader (Synergy^TM^ HT 96-well UV-Vis spectrophotometer with a Gen5 data analysis software from BioTek Instruments, Inc., Winooski, VT, USA). The percentage of inhibition was then calculated as follows:

(1) $$\%\;\mathrm{inhibition}=\left(1-\frac{B-b}{A-a}\right)\;\times \;100$$

where *A* is the initial velocity of the reaction with enzyme, *a* is the initial velocity of the reaction without enzyme, *B* is the initial velocity of the enzyme reaction with extract, and *b* is the initial velocity of the reaction with extract but without enzyme.

The BChE inhibitory activities of *Morus* fruit extracts were determined similarly to AChE, except that 100 ng equine serum BChE (≥10 units/mg protein, 100 μL) in 50 mM KPB (pH 7.0) containing 1 mM MgCl_2_ and 0.1 mM butyrylthiocholine (BTCh) in 50 mM KPB (pH 7.0) were used as the reaction enzyme and substrate, respectively [38,39]. All enzymes, chemicals and reagents for cholinesterase inhibitions were purchased from Sigma-Aldrich (St. Louis, MO, USA).

The BACE-1 activity was determined utilizing the fluorescence resonance energy transfer (FRET) method on a BACE-1 activity detection kit (Sigma-Aldrich). The manufacturer’s instructions were followed, and the results were expressed as a percentage of BACE-1 inhibition as above.

### 4.5. Statistical Analysis

All experiments were carried out in triplicate (*n* = 3) and expressed as mean ± standard deviation (SD). One-way Analysis of Variance (ANOVA) and Duncan’s multiple comparison tests were performed to determine the significant differences between values with *p* < 0.05. Two-way Pearson bivariated correlation test was performed to determine the significantly different correlation between values with *p* ≤ 0.01 and *p* ≤ 0.05. Mean value of all variables investigated in the samples were also subjected to principal component analysis (PCA) and agglomerative hierarchical clustering analysis (AHC) using the XLSTAT-base version 2019.3.2 software (Addinsoft, New York, NY, USA).

## Figures and Tables

**Table 1:** Quantification of total phenolic contents (TPCs), anthocyanidin (cyanidin) and anthocyanins (keracyanin and kuromanin) of aqueous extracts of *Morus* fruit samples.

Order	Cultivars	TPCs (mg GAE/g DW)	Anthocyanidin (µg/g DW)	Anthocyanins (µg/g DW)	
			Cyanidin	Keracyanin	Kuromanin
1	*Morus* ‘Krua’	3.72 ± 0.18 ^n^	347.41 ± 32.02 ^f^	883.55 ± 62.41 ^h^	1249.03 ± 108.70 ^g^
2	*Morus* ‘Jak’	2.75 ± 0.11 ^o^	45.87 ± 3.01 ^ghi^	235.19 ± 0.86 ^mn^	213.03 ± 0.90 ^mn^
3	*Morus* ‘Pai’	2.76 ± 0.10 ^o^	87.58 ± 7.58 ^ghi^	228.07 ± 2.80 ^mn^	211.58 ± 2.91 ^mn^
4	*Morus* ‘Pai-Ubon’	3.74 ± 0.17 ^n^	86.50 ± 6.15 ^ghi^	237.32 ± 0.26 ^mn^	160.36 ± 1.00 ^mno^
5	*Morus* ‘Poe’	3.73 ± 0.12 ^n^	160.11 ± 8.13 ^ghi^	522.74 ± 1.79 ^j^	905.26 ± 4.89 ^h^
6	*Morus* ‘Mae Luke On’	4.08 ± 0.39 ^m^	61.95 ± 5.00 ^ghi^	155.23 ± 2.71 ^no^	194.19 ± 1.40 ^mn^
7	*Morus* ‘Som’	5.18 ± 0.19 ^jk^	98.12 ± 4.18 ^ghi^	235.99 ± 5.41 ^mn^	172.13 ± 3.05 ^mn^
8	*Morus* ‘Som Yai’	4.10 ± 0.38 ^m^	104.06 ± 11.16 ^ghi^	299.28 ± 0.40 ^lm^	263.20 ± 1.78 ^klmn^
9	*Morus* ‘Sida’	5.58 ± 0.48 ^hi^	184.74 ± 16.86 ^gh^	630.07 ± 1.09 ^i^	814.19 ± 1.41 ^h^
10	*Morus* ‘Kun Pai’	7.87 ± 0.43 ^e^	1710.50 ± 155.12 ^bc^	2765.88 ± 42.49 ^d^	7374.67 ± 30.48 ^c^
11	*Morus* ‘Nakhon Ratchasima 60’	3.53 ± 0.20 ^n^	48.95 ± 5.08 ^ghi^	161.99 ± 0.30 ^no^	88.50 ± 0.38 ^no^
12	*Morus* ‘Buri Ram 51’	3.70 ± 0.29 ^n^	107.50 ± 8.29 ^ghi^	536.22 ± 37.80 ^j^	222.42 ± 18.22 ^mn^
13	*Morus* ‘Buri Ram 60’	4.68 ± 0.23 ^l^	68.23 ± 0.23 ^ghi^	372.89 ± 0.03 ^kl^	309.09 ± 0.78 ^klm^
14	*Morus* ‘Si Sa Ket 33’	5.45 ± 0.44 ^ij^	43.16 ± 4.92 ^hi^	498.91 ± 0.92 ^j^	405.97 ± 1.35 ^jkl^
15	*Morus* ‘Number 44’	8.84 ± 0.74 ^d^	143.25 ± 11.45 ^ghi^	1025.16 ± 5.36 ^g^	1233.66 ± 19.54 ^g^
16	*Morus* sp. code SKSM 820281	7.11 ± 0.58 ^f^	1789.96 ± 127.59 ^ab^	4874.70 ± 83.40 ^c^	6426.53 ± 156.12 ^d^
17	*Morus* sp. code SKSM 14-13-20	10.81 ± 0.21 ^b^	1583.49 ± 113.87 ^cd^	5848.59 ± 65.45 ^b^	10141.24 ± 71.43 ^b^
18	*Morus* sp. code SKSM 040691	5.05 ± 0.28 ^k^	62.07 ± 6.66 ^ghi^	0.00 ^p^	0.00 ^o^
19	*Morus* sp. code SKSM 810191	10.27 ± 0.50 ^c^	2879.30 ± 228.33 ^a^	7588.34 ± 36.59 ^a^	13566.64 ± 37.40 ^a^
20	*Morus* sp. code SKSM 810391	0.37 ± 0.01 ^p^	1502.84 ± 157.69 ^d^	2408.50 ± 159.13 ^e^	5447.20 ± 305.14 ^e^
21	*Morus* sp. code SRCM 9124-12	5.81 ± 0.19 ^h^	0.00 ^i^	403.03 ± 0.92 ^k^	560.18 ± 2.29 ^ij^
22	*Morus* sp. code SRCM 9801-465	7.20 ± 0.48 ^f^	182.72 ± 14.62 ^gh^	720.44 ± 61.74 ^i^	1363.68 ± 111.80 ^g^
23	*Morus* sp. code SRCM 9801-535	5.58 ± 0.28 ^hi^	207.84 ± 10.43 ^fg^	121.33 ± 8.63 ^o^	139.19 ± 9.84 ^mno^
24	*Morus* sp. code SRCM 9801-833	5.39 ± 0.29 ^ij^	344.58 ± 29.88 ^f^	346.69 ± 5.19 ^kl^	421.43 ± 6.22 ^jk^
25	*Morus* sp. code SRCM 9806-271	6.79 ± 0.20 ^g^	713.78 ± 25.11 e	1428.44 ± 0.07 ^f^	2634.51 ± 9.53 ^f^
26	*Morus* sp. code SRCM 9806-287	11.86 ± 0.19 ^a^	676.62 ± 41.02 ^e^	685.19 ± 11.64 ^i^	611.59 ± 8.34 ^i^
27	*Morus* sp. code SRCM 9809-34	5.55 ± 0.26 ^hi^	41.25 ± 1.68 ^hi^	152.75 ± 1.02 ^no^	247.17 ± 0.20 ^lmn^

Values expressed are mean ± standard deviation (SD) of triplicate experiments (*n* = 3). Lowercase letter indicates significant differences in each column at *p* < 0.05 calculated by one-way analysis of variance (ANOVA) and Duncan’s multiple comparison test.

**Table 2:** Antioxidant analysis of aqueous extracts of *Morus* fruit samples.

Order	Cultivars	DPPH Radical Scavenging Assay (µmol TE/100 g DW)	FRAP Assay (µmol TE/g DW)	ORAC Assay (µmol TE/g DW)
1	*Morus* ‘Krua’	0.62 ± 0.05 ^e^	4.39 ± 0.18 ^m^	201.81 ± 15.60 ^cd^
2	*Morus* ‘Jak’	0.50 ± 0.05 ^gh^	2.45 ± 0.19 ^n^	134.19 ± 11.06 ^gh^
3	*Morus* ‘Pai’	0.49 ± 0.05 ^h^	2.30 ± 0.17 ^n^	151.04 ± 13.62 ^fg^
4	*Morus* ‘Pai-Ubon’	0.56 ± 0.05 ^f^	2.61 ± 0.11 ^mn^	172.53 ± 15.75 ^ef^
5	*Morus* ‘Poe’	0.58 ± 0.05 ^f^	3.33 ± 0.16 ^mn^	151.16 ± 12.03 ^fg^
6	*Morus* ‘Mae Luke On’	0.45 ± 0.04 ^h^	14.24 ± 0.84 ^jk^	130.52 ± 12.01 ^ghi^
7	*Morus* ‘Som	0.45 ± 0.04 ^h^	18.50 ± 0.79 ^h^	254.04 ± 23.15 ^b^
8	*Morus* ‘Som Yai’	0.50 ± 0.04 ^gh^	13.32 ± 0.63 ^k^	91.92 ± 8.78 ^klm^
9	*Morus* ‘Sida’	0.54 ± 0.05 ^fg^	16.55 ± 1.43 ^i^	112.16 ± 9.24 ^hijk^
10	*Morus* ‘Kun Pai’	1.25 ± 0.02 ^a^	44.33 ± 0.76 ^d^	216.42 ± 53.27 ^c^
11	*Morus* ‘Nakhon Ratchasima 60’	0.28 ± 0.02 ^k^	11.02 ± 0.54 ^l^	131.73 ± 12.37 ^ghi^
12	*Morus* ‘Buri Ram 51’	0.32 ± 0.03 ^j^	11.37 ± 0.47 ^l^	103.07 ± 6.55 ^jkl^
13	*Morus* ‘Buri Ram 60’	0.37 ± 0.03 ^i^	15.50 ± 0.90 ^ij^	158.20 ± 11.01 ^f^
14	*Morus* ‘Si Sa Ket 33’	0.37 ± 0.01 ^i^	15.56 ± 1.33 ^ij^	178.98 ± 17.51 ^e^
15	*Morus* ‘Number 44’	0.83 ± 0.08 ^b^	27.90 ± 1.10 ^g^	251.84 ± 21.98 ^b^
16	*Morus* sp. code SKSM 820281	0.50 ± 0.03 ^gh^	43.28 ± 2.85 ^d^	192.66 ± 48.44 ^de^
17	*Morus* sp. code SKSM 14-13-20	0.72 ± 0.05 ^c^	66.96 ± 6.48 ^b^	259.25 ± 40.32 ^b^
18	*Morus* sp. code SKSM 040691	0.67 ± 0.06 ^d^	14.70 ± 0.20 ^ijk^	86.63 ± 7.72 ^lm^
19	*Morus* sp. code SKSM 810191	0.75 ± 0.04 ^c^	63.97 ± 3.84 ^c^	283.20 ± 36.56 ^a^
20	*Morus* sp. code SKSM 810391	0.75 ± 0.07 ^c^	117.87 ± 1.77 ^a^	109.74 ± 3.64 ^ijk^
21	*Morus* sp. code SRCM 9124-12	0.73 ± 0.06 ^c^	15.75 ± 0.53 ^ij^	64.03 ± 4.32 ^n^
22	*Morus* sp. code SRCM 9801-465	0.55 ± 0.03 ^f^	3.06 ± 0.23 ^mn^	77.71 ± 3.84 ^mn^
23	*Morus* sp. code SRCM 9801-535	0.56 ± 0.05 ^f^	16.64 ± 3.17 ^i^	103.79 ± 8.10 ^jkl^
24	*Morus* sp. code SRCM 9801-833	0.55 ± 0.04 ^f^	14.20 ± 0.71 ^jk^	113.79 ± 9.44 ^hijk^
25	*Morus* sp. code SRCM 9806-271	0.67 ± 0.06 ^d^	30.15 ± 1.52 ^f^	151.52 ± 11.82 ^fg^
26	*Morus* sp. code SRCM 9806-287	0.85 ± 0.07 ^b^	40.52 ± 1.16 ^e^	116.96 ± 9.64 ^hij^
27	*Morus* sp. code SRCM 9809-34	0.55 ± 0.03 ^f^	15.28 ± 1.34 ^ij^	82.85 ± 5.84 ^lmn^

Values expressed are mean ± standard deviation (SD) of triplicate experiments (*n* = 3). Lowercase letter indicates significant differences in each column at *p* < 0.05 calculated by one-way analysis of variance (ANOVA) and Duncan’s multiple comparison test.

**Table 3:** Anti-Alzheimer properties of aqueous extracts of *Morus* fruit samples towards inhibitions of acetylcholinesterase (AChE), butyrylcholinesterase (BChE), and beta-secretase 1 (BACE-1).

Order	Cultivars	Percentage of Inhibition (%)		
		AChE	BChE	BACE-1
1	*Morus* ‘Krua’	44.91 ± 2.62 ^f^	42.38 ± 2.35 ^j^	41.58 ± 7.89 ^j^
2	*Morus* ‘Jak’	37.35 ± 1.54 ^ij^	51.16 ± 4.58 ^g^	58.61 ± 0.41 ^efg^
3	*Morus* ‘Pai’	34.68 ± 3.11 ^jkl^	51.30 ± 3.62 ^g^	38.78 ± 2.26 ^j^
4	*Morus* ‘Pai-Ubon’	37.09 ± 3.44 ^ijk^	48.36 ± 3.63 ^gh^	71.23 ± 0.00 ^bc^
5	*Morus* ‘Poe’	33.70 ± 3.31 ^lm^	55.34 ± 1.05 ^f^	37.48 ± 2.07 ^jk^
6	*Morus* ‘Mae Luke On’	45.71 ± 3.95 ^ef^	56.52 ± 5.16 ^ef^	51.27 ± 3.32 ^ghi^
7	*Morus* ‘Som’	56.29 ± 2.41 ^b^	77.02 ± 3.14 ^a^	63.01 ± 5.22 ^de^
8	*Morus* ‘Som Yai’	53.89 ± 4.20 ^bc^	63.16 ± 1.03 ^c^	63.00 ± 7.50 ^de^
9	*Morus* ‘Sida’	49.65 ± 2.53 ^d^	64.86 ± 2.39 ^c^	52.81 ± 1.37 ^ghi^
10	*Morus* ‘Kun Pai’	35.37 ± 1.77 ^jkl^	41.78 ± 1.28 ^j^	48.93 ± 1.66 ^i^
11	*Morus* ‘Nakhon Ratchasima 60’	60.09 ± 3.62 ^a^	62.72 ± 5.23 ^c^	54.21 ± 2.69 ^ghi^
12	*Morus* ‘Buri Ram 51’	53.66 ± 3.40 ^bc^	71.33 ± 6.79 ^b^	65.54 ± 3.44 ^cde^
13	*Morus* ‘Buri Ram 60’	45.61 ± 3.96 ^ef^	56.85 ± 1.50 ^ef^	70.65 ± 1.02 ^bc^
14	*Morus* ‘Si Sa Ket 33’	45.85 ± 4.47 ^ef^	62.99 ± 3.03 ^c^	76.32 ± 2.06 ^ab^
15	*Morus* ‘Number 44’	50.98 ± 2.73 ^cd^	61.31 ± 3.53 ^cd^	70.45 ± 3.54 ^bc^
16	*Morus* sp. code SKSM 820281	34.05 ± 3.70 ^klm^	48.76 ± 4.59 ^gh^	55.12 ± 1.38 ^fghi^
17	*Morus* sp. code SKSM 14-13-20	31.37 ± 2.25 ^m^	37.80 ± 3.79 ^k^	77.11 ± 5.60 ^ab^
18	*Morus* sp. code SKSM 040691	26.10 ± 1.44 ^n^	43.86 ± 0.91 ^ij^	54.60 ± 3.59 ^fghi^
19	*Morus* sp. code SKSM 810191	43.68 ± 2.28 ^fg^	59.05 ± 2.28 ^de^	66.34 ± 2.06 ^cd^
20	*Morus* sp. code SKSM 810391	21.87 ± 1.48 ^o^	22.02 ± 2.20 ^m^	61.64 ± 6.11 ^def^
21	*Morus* sp. code SRCM 9124-12	48.52 ± 4.78 ^de^	50.57 ± 2.22 ^gh^	31.28 ± 1.78 ^k^
22	*Morus* sp. code SRCM 9801-465	22.96 ± 1.42 ^o^	21.27 ± 1.96 ^m^	58.10 ± 3.23 ^efgh^
23	*Morus* sp. code SRCM 9801-535	39.98 ± 2.72 ^hi^	61.63 ± 3.07 ^cd^	51.03 ± 0.36 ^hi^
24	*Morus* sp. code SRCM 9801-833	36.50 ± 2.07 ^jkl^	48.75 ± 1.48 ^gh^	62.85 ± 0.97 ^de^
25	*Morus* sp. code SRCM 9806-271	41.21 ± 3.00 ^gh^	51.87 ± 4.24 ^g^	76.12 ± 5.45 ^ab^
26	*Morus* sp. code SRCM 9806-287	26.63 ± 2.20 ^n^	30.00 ± 2.60 ^l^	78.67 ± 5.70 ^a^
27	*Morus* sp. code SRCM 9809-34	36.17 ± 3.55 ^jkl^	47.06 ± 3.21 ^hi^	64.79 ± 2.52 ^cde^

Values expressed are mean ± standard deviation (SD) of triplicate experiments (*n* = 3). Lowercase letter indicates significant differences in each column at *p* < 0.05 calculated by one-way analysis of variance (ANOVA) and Duncan’s multiple comparison test. The extract concentration was 5 mg/mL in all enzyme assays.

**Table 4:** Correlation coefficient (*r*) of total phenolic contents (TPCs), cyanidin contents, keracyanin contents, kuromanin contents, antioxidant activities as being determined by 2,2-diphenyl-1-picrylhydrazyl (DPPH) radical scavenging, ferric ion reducing antioxidant power (FRAP), and oxygen radical absorbance capacity (ORAC) assays and anti-Alzheimer activities through inhibition of the key enzymes (acetylcholinesterase (AChE), butyrylcholinesterase (BChE), and beta-secretase 1 (BACE-1)) of aqueous extracts of *Morus* fruit samples.

Parameters	TPCs	Cyanidin	Keracyanin	Kuromanin	DPPH	FRAP	ORAC	Anti-AChE	Anti-BChE	Anti-BACE1
**TPCs**	1									
**Cyanidin**	0.476 *	1								
**Keracyanin**	0.540 **	0.943 **	1							
**Kuromanin**	0.505 **	0.963 **	0.977 **	1						
**DPPH**	0.502 **	0.527 **	0.381	0.498 **	1					
**FRAP**	0.242	0.772 **	0.678 **	0.725 **	0.481 *	1				
**ORAC**	0.421 *	0.543 **	0.626 **	0.610 **	0.269	0.300	1			
**Anti-AChE**	−0.138	−0.305	−0.204	−0.247	−0.418 *	−0.360	0.178	1		
**Anti-BChE**	−0.154	−0.297	−0.193	−0.252	−0.480 *	−0.413 *	0.208	0.860 **	1	
**Anti-BACE1**	0.416 *	0.178	0.213	0.175	−0.350	0.300	0.269	−0.037	−0.020	1

** Correlation is significant at *p* ≤ 0.01 (2-tailed bivariated correlation). * Correlation is significant at *p* ≤ 0.05 (2-tailed bivariated correlation).

**Table 5:** Solvent system of anthocyanin analysis using HPLC analysis.

Time (min)	Solvent A	Solvent B
0	88	12
6	88	12
8	85	15
25	85	15
25	88	12
30	88	12

Solvent A = Milli-Q water containing 0.4% (*v*/*v*) TFA; solvent B = acetonitrile containing 0.4% (*v*/*v*) TFA.

## Supplementary Materials

Supplementary Table S1: Images of twenty-seven cultivars, sample codes, and collectors of *Morus* fruit samples used in this experiment. Supplementary Table S2: Color (where L describes darkness (−) to lightness (+), a describes green (−) to red (+) colors, and b describes indigo (−) to yellow (+) colors) and the percentage (%) of moisture content of fresh and freeze-dried *Morus* fruit samples. Supplementary Figure S1: High-performance liquid chromatography (HPLC) chromatograms of (A.) cyanidin chloride standard, and anthocyanidin analyses of *Morus* fruit extracts including (B.) *Morus* ‘Krua’, (C.) *Morus* ‘Jak’, (D.) *Morus* ‘Pai’, (E.) *Morus* ‘Pai-Ubon’, (F.) *Morus* ‘Poe’, (G.) *Morus* ‘Mae Luke On’, (H.) *Morus* ‘Som’, (I.) *Morus* ‘Som Yai’, (J.) *Morus* ‘Sida’, (K.) *Morus* ‘Kun Pai’, (L.) *Morus* ‘Nakhon Ratchasima 60’, (M.) *Morus* ‘Buri Ram 51’, (N.) *Morus* ‘Buri Ram 60’, (O.) *Morus* ‘Si Sa Ket 33’, (P.) *Morus* ‘Number 44’, (Q.) *Morus* sp. code SKSM 820281, (R.) *Morus* sp. code SKSM 14-13-20, (S.) *Morus* sp. code SKSM 040691, (T.) *Morus* sp. code SKSM 810191’, (U.) *Morus* sp. code SKSM 810391, (V.) *Morus* sp. code SRCM 9124-12, (W.) *Morus* sp. code SRCM 9801-465, (X.) *Morus* sp. code SRCM 9801-535, (Y.) *Morus* sp. code SRCM 9801-833, (Z.) *Morus* sp. code SRCM 9806-271, (AA.) *Morus* sp. code SRCM 9806-287, and (AB.) *Morus* sp. code SRCM 9809-34. The retention times (*R_t_*) of cyanidin chloride in *Morus* fruit extracts were also indicated. Supplementary Figure S2: High-performance liquid chromatography (HPLC) chromatograms of (A.) kuromanin and keracyanin standards, and anthocyanin analyses of *Morus* fruit extracts including (B.) *Morus* ‘Krua’, (C.) *Morus* ‘Jak’, (D.) *Morus* ‘Pai’, (E.) *Morus* ‘Pai-Ubon’, (F.) *Morus* ‘Poe’, (G.) *Morus* ‘Mae Luke On’, (H.) *Morus* ‘Som’, (I.) *Morus* ‘Som Yai’, (J.) *Morus* ‘Sida’, (K.) *Morus* ‘Kun Pai’, (L.) *Morus* ‘Nakhon Ratchasima 60’, (M.) *Morus* ‘Buri Ram 51’, (N.) *Morus* ‘Buri Ram 60’, (O.) *Morus* ‘Si Sa Ket 33’, (P.) *Morus* ‘Number 44’, (Q.) *Morus* sp. code SKSM 820281, (R.) *Morus* sp. code SKSM 14-13-20, (S.) *Morus* sp. code SKSM 040691, (T.) *Morus* sp. code SKSM 810191’, (U.) *Morus* sp. code SKSM 810391, (V.) *Morus* sp. code SRCM 9124-12, (W.) *Morus* sp. code SRCM 9801-465, (X.) *Morus* sp. code SRCM 9801-535, (Y.) *Morus* sp. code SRCM 9801-833, (Z.) *Morus* sp. code SRCM 9806-271, (AA.) *Morus* sp. code SRCM 9806-287, and (AB.) *Morus* sp. code SRCM 9809-34. The retention times (*R_t_*) of kuromanin and keracyanin in *Morus* fruit extracts were also indicated.

## Fundings

This study was supported by Biodiversity-Based Economy Development Office (BEDO) (grant No. 22/2561), and partially supported by Grants-in-Aid from JSPS Core to-Core Program B. Asia-Africa Science Platforms
