# Supplementary material for: Comparison of Phytochemicals, Antioxidant, and In Vitro Anti-Alzheimer Properties of Twenty-Seven Morus spp. Cultivated in Thailand
Source: Molecules. 2020 Jun 3;25(11):2600. doi: 10.3390/molecules25112600 (PMC7321130; doi:10.3390/molecules25112600)

molecules

Supplementary materials

Comparison of Phytochemicals, Antioxidant, and In Vitro Anti-Alzheimer Properties of Twenty-seven *Morus* spp. Cultivated in Thailand

Piya Temviriyanukul[1], Varittha Sritalahareuthai[1], Kriskamol Na Jom[2], Butsara Jongruaysup[3], Somying Tabtimsri[4], Kanchana Pruesapan[5], Sirinapa Thangsiri[1], Woorawee Inthachat[1], Dalad Siriwan[6], Somsri Charoenkiatkul[1] and Uthaiwan Suttisansanee[1,*]

[1] Institute of Nutrition, Mahidol University, Salaya, Phuttamonthon, Nakhon Pathom 73170, Thailand; piya.tem@mahidol.ac.th (P.T.); varittha.sri@hotmail.com (V.S.); poo.sweet@hotmail.com (S.Th.); woorawee.int@mahidol.ac.th (W.I.); somsri.chr@mahidol.ac.th (S.C.); uthaiwan.sut@mahidol.ac.th (U.S.)
[2] Department of Food Science and Technology, Faculty of Agro-Industry, Kasetsart University, Chatuchak, Bangkok 10900, Thailand; kriskamol.n@ku.ac.th
[3] Office of Sericulture Conservation and Standard Conformity Assessment, The Queen Sirikit Department of Sericulture, Ministry of Agriculture and Cooperatives, Bangkok 10900, Thailand; butsara_2000@hotmail.com
[4] The Queen Sirikit Department of Sericulture Center (Kanchanaburi), Nong Ya, Mueang Kanchanaburi District, Kanchanaburi, 71000; yodyingtts@gmail.com
[5] Plant Varieties Protection Office, Department of Agriculture, Ministry of Agriculture and Cooperatives, Bangkok 10900, Thailand; kpruesapan@gmail.com
[6] Institute of Food Research and Product Development, Kasetsart University, Chatuchak, Bangkok 10900, Thailand; dalad.s@ku.th
* Correspondence: uthaiwan.sut@mahidol.ac.th; Tel.: +662-800-2380 ext. 422

Supplementary Table S1:

Images of twenty-seven cultivars, sample codes, and collectors of *Morus* fruit samples used in this experiment.

Order	Scientific name/ Cultivars/Sample code/Collector	Physical appearance	Size (cm)
1	*Morus* sp./ *Morus* 'Krua'/ KRI-2/ Somying Tabtimsri	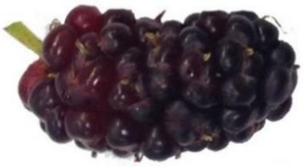	1.5
2	*Morus* sp. / *Morus* 'Jak' / KRI-3 / Somying Tabtimsri	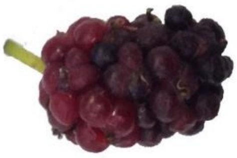	1.1
3	*Morus rotundiloba* / *Morus* 'Pai' / KRI-4 / Somying Tabtimsri	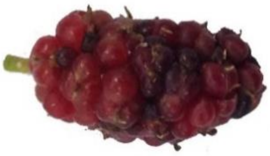	1.2
4	*Morus rotundiloba* / *Morus* 'Pai-Ubon' / KRI-5 / Somying Tabtimsri	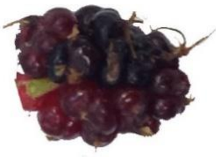	1.0
5	*Morus rotundiloba* / *Morus* 'Poe' / KRI-6 / Somying Tabtimsri	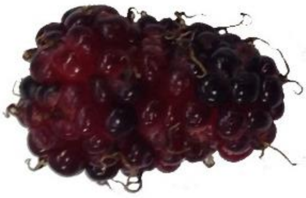	1.4
6	*Morus rotundiloba* / *Morus* 'Mae Luke On' / KRI-7 / Somying Tabtimsri	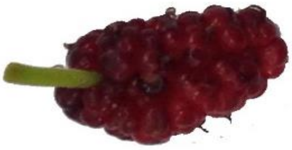	1.3
7	*Morus rotundiloba* / *Morus* 'Som' / KRI-8 / Somying Tabtimsri	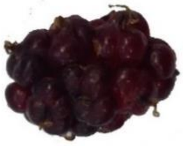	0.9

Every *Morus* fruit samples are under the same scale (———————— = 1 cm.)

Supplementary Table S1 (Cont.):

Images of twenty-seven cultivars, sample codes, and collectors of *Morus* fruit samples used in this experiment.

Order	Scientific name/ Cultivars/Sample code/Collector	Physical appearance	Size (cm)
8	*Morus* sp. / *Morus* 'Som Yai' / KRI-8 / Somying Tabtimsri	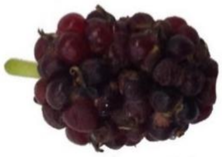	1.0
9	*Morus* sp. / *Morus* 'Sida' / KRI-10 / Somying Tabtimsri	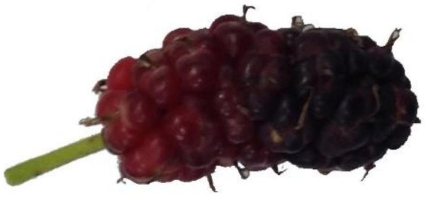	1.7
10	*Morus* sp. / *Morus* 'Kun Pai' / KRI-1 / Somying Tabtimsri	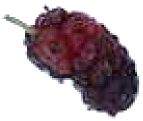	0.6
11	*Morus* sp. / *Morus* 'Nakhon Ratchasima 60' / KRI-11 / Somying Tabtimsri	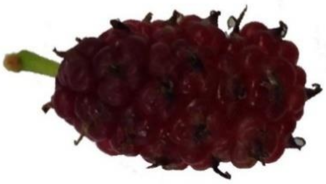	1.4
12	*Morus* sp. / *Morus* 'Buri Ram 51' / KRI-13 / Somying Tabtimsri	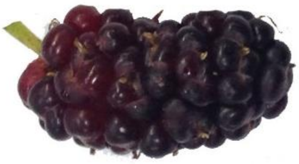	1.5
13	*Morus* sp. / *Morus* 'Buri Ram 60' / KRI-12 / Somying Tabtimsri	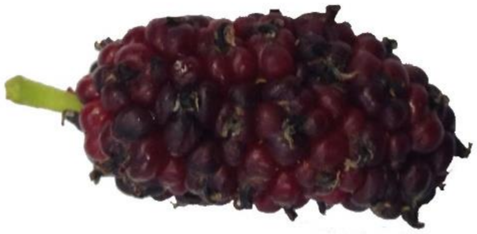	2.3

Every *Morus* fruit samples are under the same scale (——————— = 1 cm.)

Supplementary Table S1 (Cont.):

Images of twenty-seven cultivars, sample codes, and collectors of *Morus* fruit samples used in this experiment.

Order	Scientific name/ Cultivars/Sample code/Collector	Physical appearance	Size (cm)
14	*Morus* sp. / *Morus* 'Si Sa Ket 33' / KRI-14 / Somying Tabtimsri	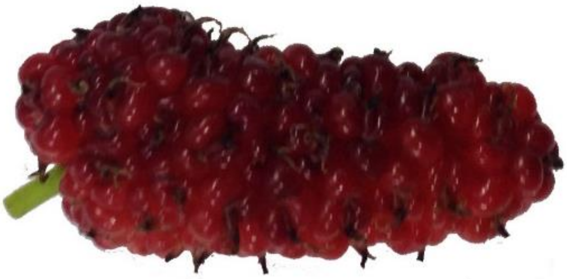	3.1
15	*Morus* sp. / *Morus* 'Number 44' / KRI-19 / Somying Tabtimsri	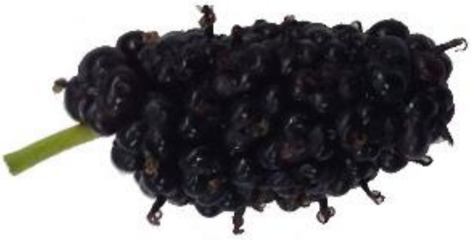	2.2
16	*Morus alba* L. / *Morus* sp. code SKSM 820281 / KRI-18 / Somying Tabtimsri	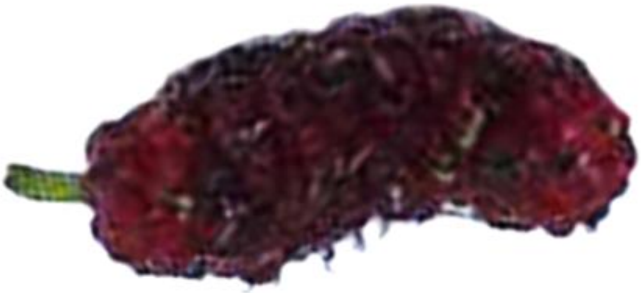	3.7
17	*Morus multicaulis* Perr. / *Morus* sp. code SKSM 14-13-20 / KRI-23 / Somying Tabtimsri	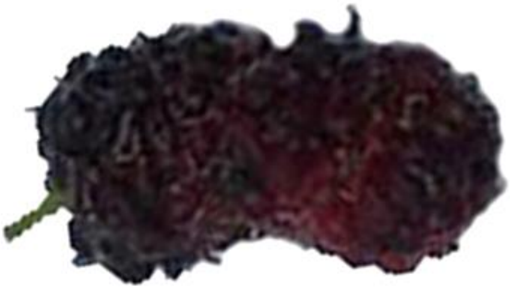	3.5

Every *Morus* fruit samples are under the same scale (——————— = 1 cm.)

Supplementary Table S1 (Cont.):

Images of twenty-seven cultivars, sample codes, and collectors of *Morus* fruit samples used in this experiment.

Order	Scientific name/ Cultivars/Sample code/Collector	Physical appearance	Size (cm)
18	*Morus* sp. / *Morus* sp. code SKSM 040691 / KRI-20 / Somying Tabtimsri	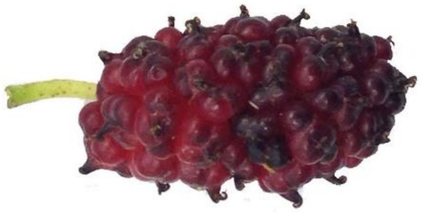	1.8
19	*Morus* sp. / *Morus* sp. code SKSM 810191 / KRI-16 / Somying Tabtimsri	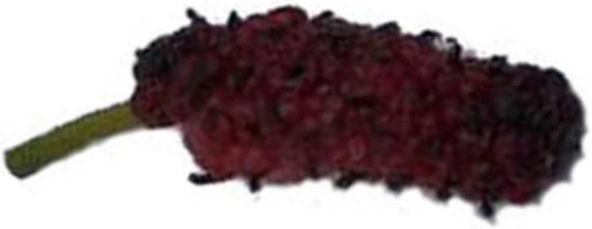	2.4
20	*Morus indica* L. / *Morus* sp. code SKSM 810391 / KRI-17 / Somying Tabtimsri	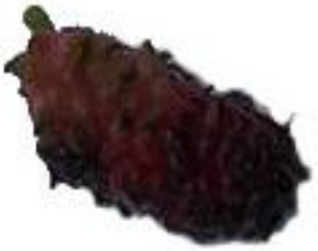	1.7
21	*Morus* sp. / *Morus* sp. code SRCM 9124-12 / KRI-24 / Somying Tabtimsri	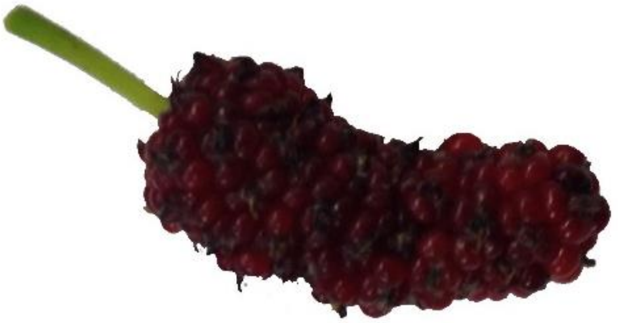	3.1
22	*Morus* sp. / *Morus* sp. code SRCM 9801-465 / KRI-25 / Somying Tabtimsri	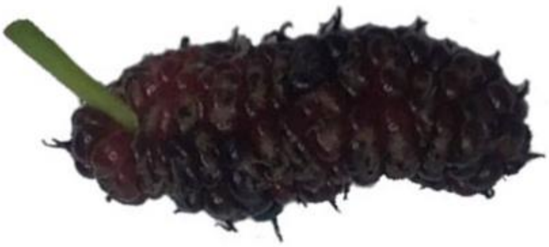	2.9

Every *Morus* fruit samples are under the same scale (———————— = 1 cm.)

Supplementary Table S1 (Cont.):

Images of twenty-seven cultivars, sample codes, and collectors of *Morus* fruit samples used in this experiment.

Order	Scientific name/ Cultivars/Sample code/Collector	Physical appearance	Size (cm)
23	*Morus* sp. / *Morus* sp. code SRCM 9801-535 / KRI-26 / Somying Tabtimsri	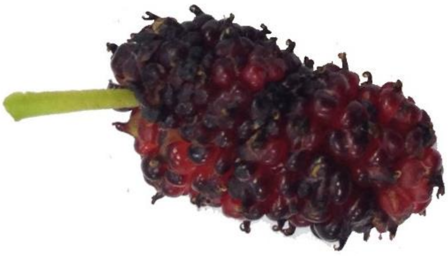	1.9
24	*Morus* sp. / *Morus* sp. code SRCM 9801-833 / KRI-27 / Somying Tabtimsri	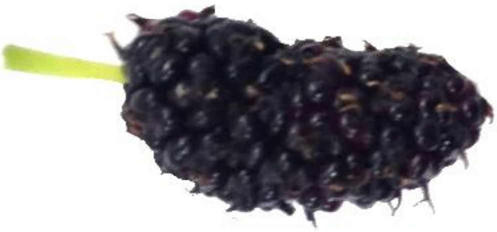	2.1
25	*Morus* sp. / *Morus* sp. code SRCM 9806-271 / KRI-28 / Somying Tabtimsri	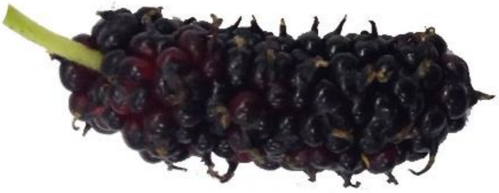	2.3
26	*Morus* sp. / *Morus* sp. code SRCM 9806-287 / KRI-29 / Somying Tabtimsri	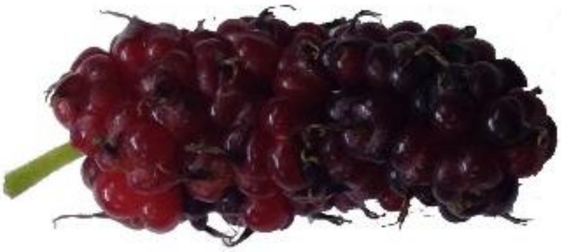	2.9
27	*Morus* sp. / *Morus* sp. code SRCM 9809-34 / KRI-30 / Somying Tabtimsri	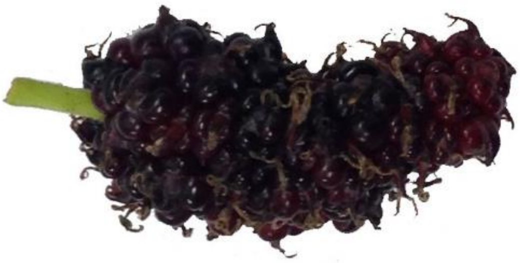	2.4

Every *Morus* fruit samples are under the same scale (———————— = 1 cm.)

Supplementary Table S2:

Color (where L describes darkness (−) to lightness (+), a describes green (−) to red (+) colors, and b describes indigo (−) to yellow (+) colors) and the percentage (%) of moisture content of fresh and freeze-dried *Morus* fruit samples

Order	Cultivars	$Color of fresh samples			$Color of dried samples			Moisture content (%) of fresh sample	Moisture content (%) of dried sample
		L	a	b	L	a	b		
1	*Morus* 'Krua'	27.35 ± 1.52	14.12 ± 1.16	17.12 ± 1.05	19.96 ± 0.02	7.73 ± 0.02	5.78 ± 0.03	79.33	6.57
2	*Morus* 'Jak'	25.35 ± 1.01	17.40 ± 0.57	15.49 ± 0.89	21.89 ± 0.02	5.97 ± 0.04	7.70 ± 0.01	82.11	5.48
3	*Morus* 'Pai'	24.20 ± 1.05	14.16 ± 2.35	14.64 ± 0.83	21.22 ± 0.06	4.98 ± 0.03	7.34 ± 0.07	77.08	5.85
4	*Morus* 'Pai-Ubon'	21.84 ± 0.77	17.52 ± 0.77	13.57 ± 0.89	22.85 ± 0.03	5.71 ± 0.01	7.76 ± 0.02	81.53	6.45
5	*Morus* 'Poe'	20.60 ± 2.71	16.72 ± 1.99	11.22 ± 1.87	19.93 ± 0.06	6.45 ± 0.01	5.66 ± 0.03	80.43	7.20
6	*Morus* 'Mae Luke On'	23.41 ± 0.72	15.15 ± 0.10	13.32 ± 0.51	21.09 ± 0.03	4.54 ± 0.03	7.32 ± 0.02	79.24	7.00
7	*Morus* 'Som'	26.58 ± 1.31	18.63 ± 0.87	16.06 ± 0.89	22.29 ± 0.01	5.95 ± 0.01	7.89 ± 0.02	77.92	5.49
8	*Morus* 'Som Yai'	23.48 ± 2.52	17.08 ± 2.33	13.79 ± 1.55	21.53 ± 0.02	6.40 ± 0.02	7.19 ± 0.02	79.30	6.15
9	*Morus* 'Sida'	24.96 ± 1.21	15.20 ± 1.16	16.01 ± 0.80	20.54 ± 0.04	7.14 ± 0.02	6.24 ± 0.03	80.69	4.09
10	*Morus* 'Kun Pai'	13.47 ± 1.45	5.44 ± 0.19	2.08 ± 0.50	21.90 ± 0.03	4.34 ± 0.03	1.00 ± 0.01	81.19	7.83
11	*Morus* 'Nakhon Ratchasima 60'	29.14 ± 1.88	17.79 ± 0.86	16.95 ± 1.00	24.73 ± 0.02	5.86 ± 0.02	7.77 ± 0.03	80.85	8.02
12	*Morus* 'Buri Ram 51'	23.35 ± 1.99	18.44 ± 0.10	13.86 ± 1.88	20.98 ± 0.04	6.36 ± 0.03	7.11 ± 0.03	86.22	6.66
13	*Morus* 'Buri Ram 60'	19.20 ± 1.09	21.49 ± 1.49	10.90 ± 0.68	26.21 ± 0.02	5.82 ± 0.01	5.48 ± 0.01	85.94	9.67
14	*Morus* 'Si Sa Ket 33'	21.55 ± 1.36	21.89 ± 2.73	13.65 ± 0.95	21.48 ± 0.05	7.44 ± 0.02	6.22 ± 0.02	83.19	9.02

Supplementary Table S2 (Cont.):

Color (where L describes darkness (−) to lightness (+), a describes green (−) to red (+) colors, and b describes indigo (−) to yellow (+) colors) and the percentage (%) of moisture content of fresh and freeze-dried *Morus* fruit samples

Order	Cultivars	$Color of fresh samples			$Color of dried samples			Moisture content (%) of fresh sample	Moisture content (%) of dried sample
		L	a	b	L	a	b		
15	*Morus* 'Number 44'	21.24 ± 4.45	17.39 ± 1.54	12.58 ± 2.59	21.33 ± 0.02	7.06 ± 0.02	4.66 ± 0.03	85.94	3.14
16	*Morus* sp. code SKSM 820281	13.09 ± 1.36	11.87 ± 0.44	4.14 ± 0.40	21.83 ± 0.03	4.73 ± 0.03	1.43 ± 0.02	86.08	8.35
17	*Morus* sp. code SKSM 14-13-20	13.97 ± 1.21	3.50 ± 0.50	0.83 ± 0.17	21.37 ± 0.06	3.31 ± 0.03	0.41 ± 0.01	85.80	9.76
18	*Morus* sp. code SKSM 040691	28.5 ± 5.58	14.36 ± 1.64	15.43 ± 3.50	22.90 ± 0.01	4.74 ± 0.03	7.61 ± 0.02	92.34	4.48
19	*Morus* sp. code SKSM 810191	9.73 ± 0.06	6.81 ± 0.27	4.80 ± 0.21	21.96 ± 0.02	6.17 ± 0.07	0.92 ± 0.02	84.32	7.15
20	*Morus* sp. code SKSM 810391	17.13 ± 0.33	10.79 ± 0.87	5.05 ± 0.42	21.42 ± 0.03	4.52 ± 0.03	0.67 ± 0.04	85.08	8.02
21	*Morus* sp. code SRCM 9124-12	25.13 ± 2.31	16.63 ± 2.20	15.00 ± 1.26	21.28 ± 0.02	4.83 ± 0.03	7.53 ± 0.03	83.37	6.02
22	*Morus* sp. code SRCM 9801-465	9.00 ± 1.00	1.00 ± 0.03	0.64 ± 0.05	23.22 ± 0.04	2.50 ± 0.03	2.46 ± 0.02	84.27	7.83
23	*Morus* sp. code SRCM 9801-535	26.25 ± 2.39	18.55 ± 1.64	16.57 ± 1.88	23.69 ± 0.05	4.12 ± 0.01	8.40 ± 0.02	85.43	8.83
24	*Morus* sp. code SRCM 9801-833	23.96 ± 4.29	15.49 ± 2.08	13.37 ± 2.89	22.89 ± 0.03	4.35 ± 0.01	6.53 ± 0.01	84.28	10.46
25	*Morus* sp. code SRCM 9806-271	21.03 ± 1.83	14.32 ± 1.71	11.24 ± 1.86	17.23 ± 0.05	7.94 ± 0.03	3.95 ± 0.05	81.14	5.01
26	*Morus* sp. code SRCM 9806-287	23.67 ± 1.43	18.36 ± 3.17	13.79 ± 1.05	20.80 ± 0.03	8.53 ± 0.02	5.35 ± 0.04	85.33	9.93
27	*Morus* sp. code SRCM 9809-34	21.65 ± 0.82	15.31 ± 1.70	13.27 ± 0.69	22.36 ± 0.02	3.95 ± 0.01	7.92 ± 0.05	85.78	9.63

Supplementary Figure S1:

High-performance liquid chromatography (HPLC) chromatograms of (A.) cyanidin chloride standard, and anthocyanidin analyses of *Morus* fruit extracts including (B.) *Morus* 'Krua', (C.) *Morus* 'Jak', (D.) *Morus* 'Pai', (E.) *Morus* 'Pai-Ubon', (F.) *Morus* 'Poe', (G.) *Morus* 'Mae Luke On', (H.) *Morus* 'Som', (I.) *Morus* 'Som Yai', (J.) *Morus* 'Sida', (K.) *Morus* 'Kun Pai', (L.) *Morus* 'Nakhon Ratchasima 60', (M.) *Morus* 'Buri Ram 51', (N.) *Morus* 'Buri Ram 60', (O.) *Morus* 'Si Sa Ket 33', (P.) *Morus* 'Number 44', (Q.) *Morus* sp. code SKSM 820281, (R.) *Morus* sp. code SKSM 14-13-20, (S.) *Morus* sp. code SKSM 040691, (T.) *Morus* sp. code SKSM 810191', (U.) *Morus* sp. code SKSM 810391, (V.) *Morus* sp. code SRCM 9124-12, (W.) *Morus* sp. code SRCM 9801-465, (X.) *Morus* sp. code SRCM 9801-535, (Y.) *Morus* sp. code SRCM 9801-833, (Z.) *Morus* sp. code SRCM 9806-271, (AA.) *Morus* sp. code SRCM 9806-287, and (AB.) *Morus* sp. code SRCM 9809-34. The retention times (R_t) of cyanidin chloride in *Morus* fruit extracts were also indicated.

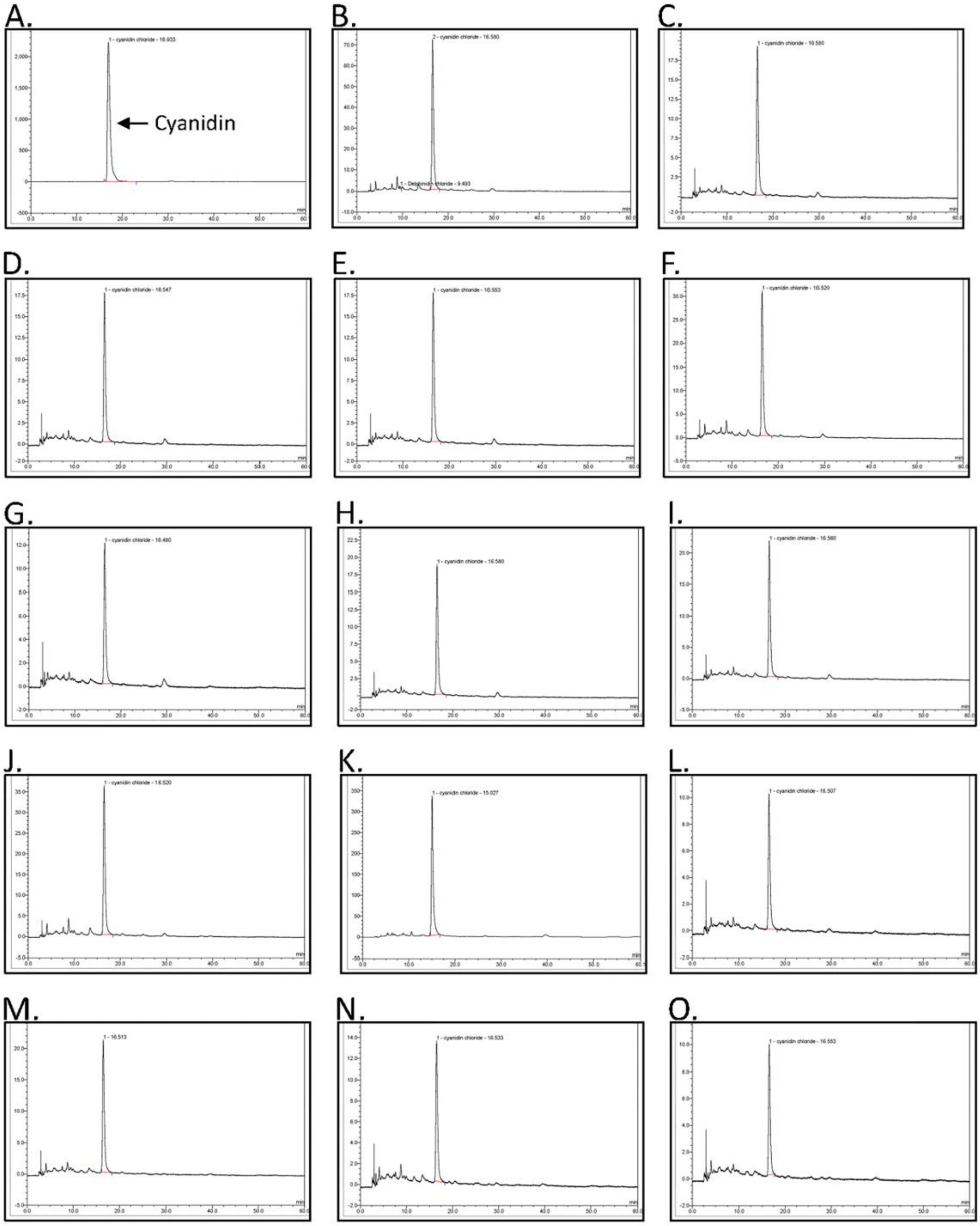

Supplementary Figure S1 (Cont.):

High-performance liquid chromatography (HPLC) chromatograms of (A.) cyanidin chloride standard, and anthocyanidin analyses of *Morus* fruit extracts including (B.) *Morus* 'Krua', (C.) *Morus* 'Jak', (D.) *Morus* 'Pai', (E.) *Morus* 'Pai-Ubon', (F.) *Morus* 'Poe', (G.) *Morus* 'Mae Luke On', (H.) *Morus* 'Som', (I.) *Morus* 'Som Yai', (J.) *Morus* 'Sida', (K.) *Morus* 'Kun Pai', (L.) *Morus* 'Nakhon Ratchasima 60', (M.) *Morus* 'Buri Ram 51', (N.) *Morus* 'Buri Ram 60', (O.) *Morus* 'Si Sa Ket 33', (P.) *Morus* 'Number 44', (Q.) *Morus* sp. code SKSM 820281, (R.) *Morus* sp. code SKSM 14-13-20, (S.) *Morus* sp. code SKSM 040691, (T.) *Morus* sp. code SKSM 810191', (U.) *Morus* sp. code SKSM 810391, (V.) *Morus* sp. code SRCM 9124-12, (W.) *Morus* sp. code SRCM 9801-465, (X.) *Morus* sp. code SRCM 9801-535, (Y.) *Morus* sp. code SRCM 9801-833, (Z.) *Morus* sp. code SRCM 9806-271, (AA.) *Morus* sp. code SRCM 9806-287, and (AB.) *Morus* sp. code SRCM 9809-34. The retention times (R_t) of cyanidin chloride in *Morus* fruit extracts were also indicated.

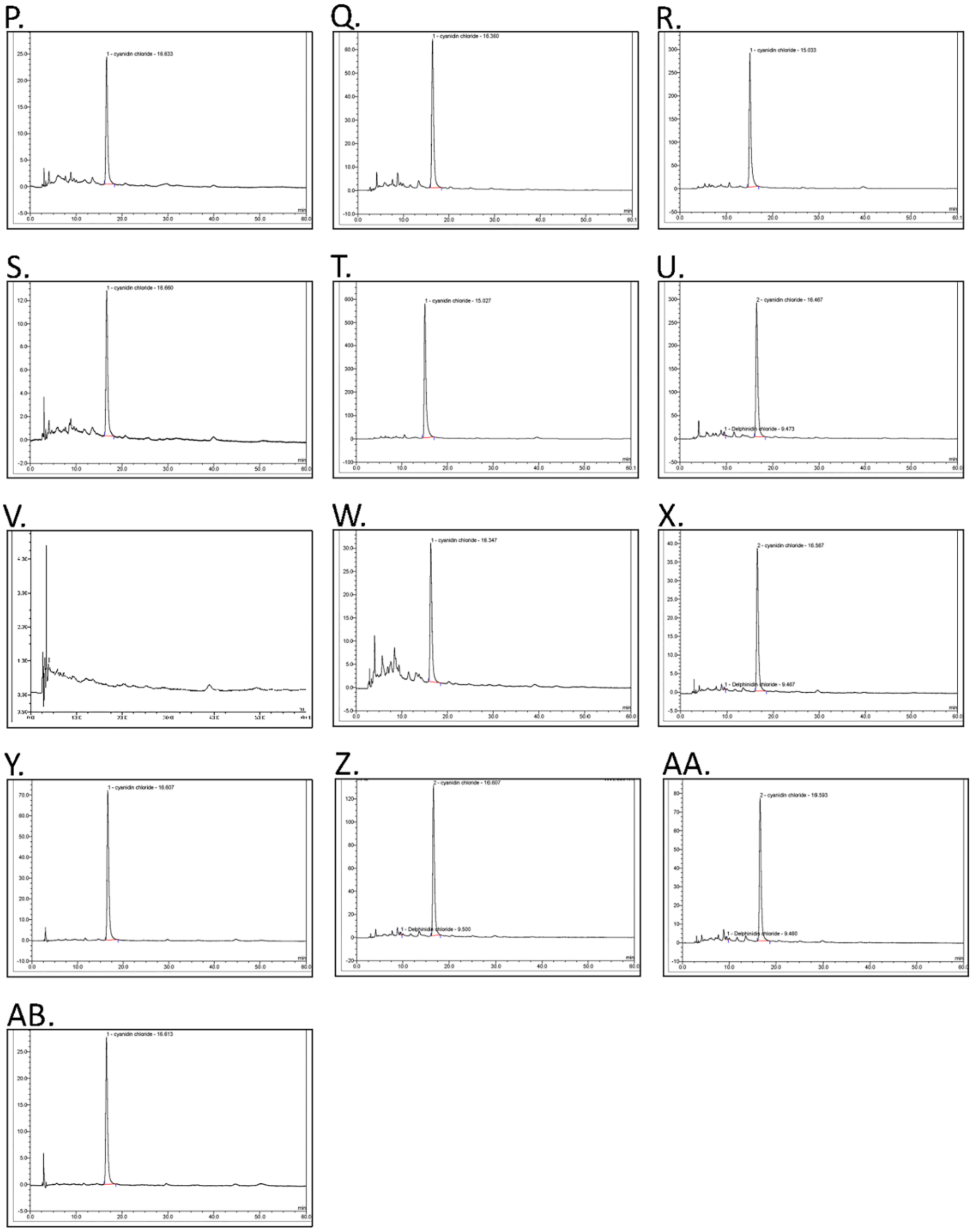

Supplementary Figure S2:

High-performance liquid chromatography (HPLC) chromatograms of (A.) kuromanin and keracyanin standards, and anthocyanin analyses of *Morus* fruit extracts including (B.) *Morus* 'Krua', (C.) *Morus* 'Jak', (D.) *Morus* 'Pai', (E.) *Morus* 'Pai-Ubon', (F.) *Morus* 'Poe', (G.) *Morus* 'Mae Luke On', (H.) *Morus* 'Som', (I.) *Morus* 'Som Yai', (J.) *Morus* 'Sida', (K.) *Morus* 'Kun Pai', (L.) *Morus* 'Nakhon Ratchasima 60', (M.) *Morus* 'Buri Ram 51', (N.) *Morus* 'Buri Ram 60', (O.) *Morus* 'Si Sa Ket 33', (P.) *Morus* 'Number 44', (Q.) *Morus* sp. code SKSM 820281, (R.) *Morus* sp. code SKSM 14-13-20, (S.) *Morus* sp. code SKSM 040691, (T.) *Morus* sp. code SKSM 810191', (U.) *Morus* sp. code SKSM 810391, (V.) *Morus* sp. code SRCM 9124-12, (W.) *Morus* sp. code SRCM 9801-465, (X.) *Morus* sp. code SRCM 9801-535, (Y.) *Morus* sp. code SRCM 9801-833, (Z.) *Morus* sp. code SRCM 9806-271, (AA.) *Morus* sp. code SRCM 9806-287, and (AB.) *Morus* sp. code SRCM 9809-34. The retention times (R_t) of kuromanin and keracyanin in *Morus* fruit extracts were also indicated.

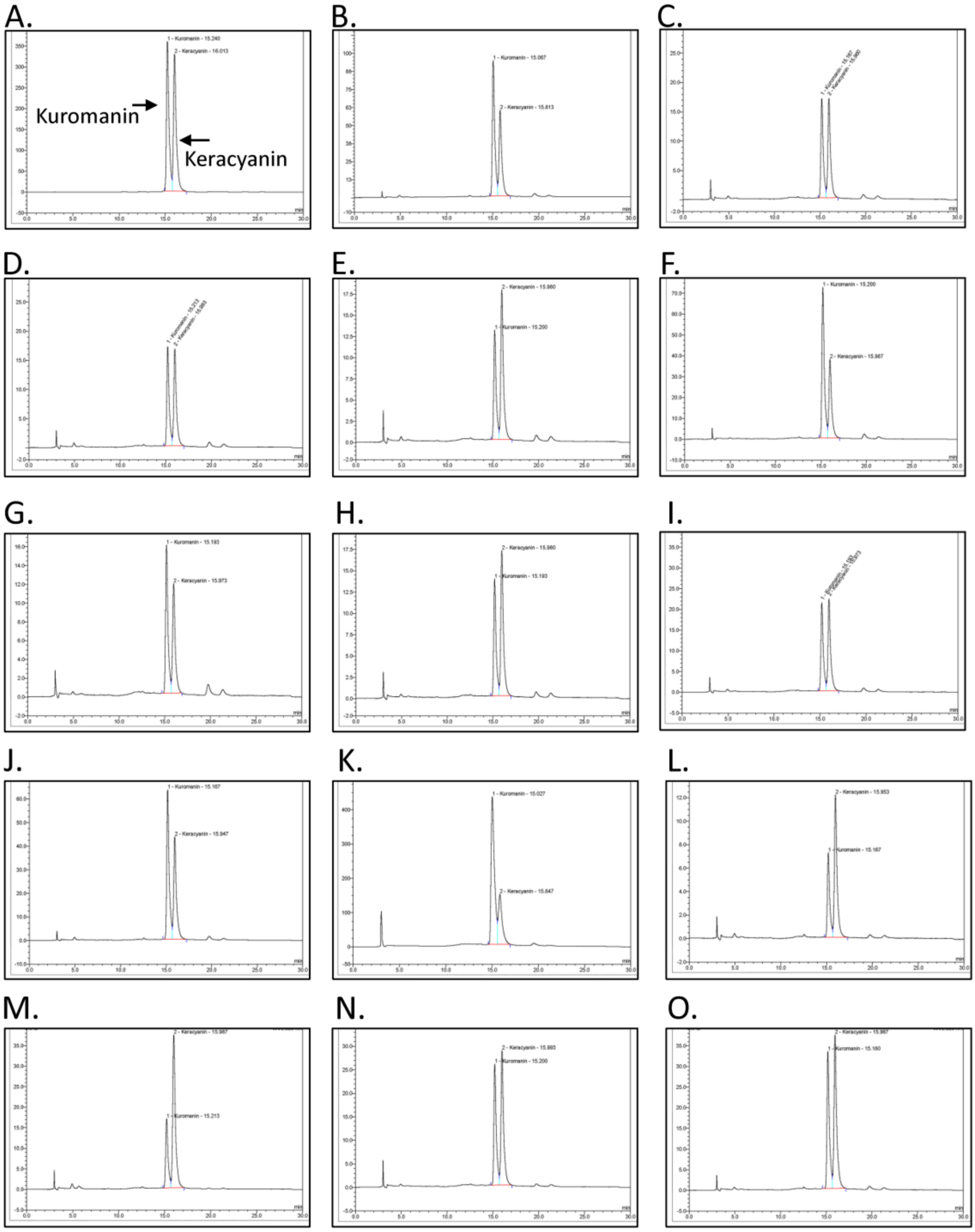

Supplementary Figure S2 (Cont.):

High-performance liquid chromatography (HPLC) chromatograms of (A.) kuromanin and keracyanin standards, and anthocyanin analyses of *Morus* fruit extracts including (B.) *Morus* 'Krua', (C.) *Morus* 'Jak', (D.) *Morus* 'Pai', (E.) *Morus* 'Pai-Ubon', (F.) *Morus* 'Poe', (G.) *Morus* 'Mae Luke On', (H.) *Morus* 'Som', (I.) *Morus* 'Som Yai', (J.) *Morus* 'Sida', (K.) *Morus* 'Kun Pai', (L.) *Morus* 'Nakhon Ratchasima 60', (M.) *Morus* 'Buri Ram 51', (N.) *Morus* 'Buri Ram 60', (O.) *Morus* 'Si Sa Ket 33', (P.) *Morus* 'Number 44', (Q.) *Morus* sp. code SKSM 820281, (R.) *Morus* sp. code SKSM 14-13-20, (S.) *Morus* sp. code SKSM 040691, (T.) *Morus* sp. code SKSM 810191', (U.) *Morus* sp. code SKSM 810391, (V.) *Morus* sp. code SRCM 9124-12, (W.) *Morus* sp. code SRCM 9801-465, (X.) *Morus* sp. code SRCM 9801-535, (Y.) *Morus* sp. code SRCM 9801-833, (Z.) *Morus* sp. code SRCM 9806-271, (AA.) *Morus* sp. code SRCM 9806-287, and (AB.) *Morus* sp. code SRCM 9809-34. The retention times (R_t) of kuromanin and keracyanin in *Morus* fruit extracts were also indicated.

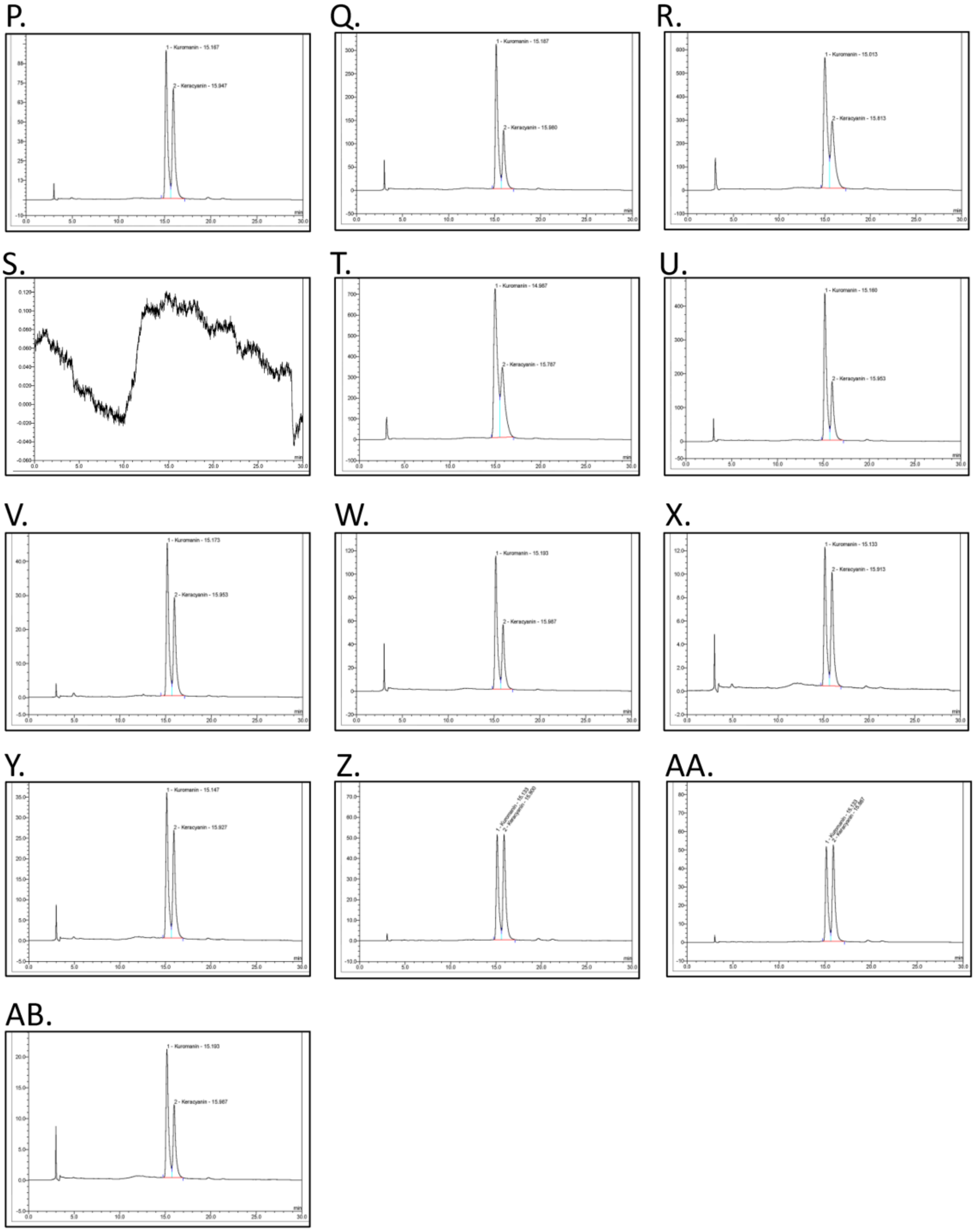

Supplement: Supplementary file 1 [file molecules-25-02600-s001.pdf]
